# Impacts of Climate Change Conditions on the Potential Distribution of *Anoplophora glabripennis* and Its Host Plants, *Salix babylonica* and *Salix matsudana*, in China

**DOI:** 10.1002/ece3.70692

**Published:** 2024-12-05

**Authors:** Liang Zhang, Ping Wang, Guanglin Xie, Wenkai Wang

**Affiliations:** ^1^ Institute of Entomology, College of Agriculture Yangtze University Jingzhou China; ^2^ MARA Key Laboratory of Sustainable Crop Production in the Middle Reaches of the Yangtze River (Co‐Construction by Ministry and Province) College of Agriculture, Yangtze University Jingzhou China

**Keywords:** *Anoplophora glabripennis*, climate change, MaxEnt model, *Salix babylonica*, *Salix matsudana*, suitable habitat

## Abstract

The *Anoplophora glabripennis* (Motschulsky) is a phytophagous pest that is seriously endangering *Salix babylonica* Linn. and *S. matsudana* Koidz. Poor control can damage local ecosystems, resulting in economic losses and management risks. In the context of climate change, the climatic ecological niche of organisms is no longer compatible with the surrounding environment. To mitigate the effects of climate change, some organisms respond adaptively to climate change through different mechanisms and in different ways. In this study, an optimized MaxEnt model was used to explore the potential distribution areas of *A. glabripennis* and its host plants, *S. babylonica* and *S. matsudana* , in response to current and future climate and to determine their movement routes and relative dynamics. The results show that the optimized model exhibits the lowest complexity and excellent prediction accuracy. It is important to note that both temperature and precipitation are the main environmental factors affecting the distribution of suitable habitats for *A. glabripennis* and its host plants. This is evidenced by the mean temperature of the warmest quarter and precipitation of the wettest month being the main environmental factors affecting the distribution of suitable habitats for the host plants. Similarly, the minimum temperature of the coldest month and precipitation seasonality are the primary bioclimatic variables constraining the dispersal of *A. glabripennis* . Under climate change, the suitable areas of both *S. babylonica* and *S. matsudana* are declining, while the suitable areas of *A. glabripennis* are expanding in future climates. Furthermore, three species exhibited a proclivity for migration to higher latitudes in response to climate change. In conclusion, this study contributes to our understanding of the biogeographic characteristics of these *A. glabripennis* , *S. babylonica* , and *S. matsudana* and provides a basis for the formulation of timely conservation strategies to reduce the potential impacts of climate change. This is of great significance for the rational management, utilization, and protection of forest ecosystems in China.

## Graphical abstract

In this study, we aimed to investigate the potential distribution areas of *A. glabripennis* and its host plants, *S. babylonica* and *S. matsudana* , in response to current and future climate conditions in China. By utilizing an optimized MaxEnt model, we were able to identify the main environmental factors influencing the distribution of these species and predict their movement routes and dynamics under changing climatic conditions. Our findings demonstrate that both temperature and precipitation play crucial roles in shaping the suitable habitats for *A. glabripennis* and its host plants. We observed a contraction of suitable areas for *S. babylonica* and *S. matsudana* , while the suitable areas for *A. glabripennis* are expanding in response to climate change. Additionally, all three species showed a tendency to migrate to higher latitudes.

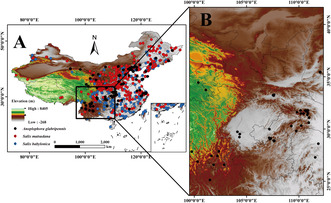

## 1 Introduction

The consequences of climate change on global ecosystems are multifaceted and intricate. In particular, changes in temperature and precipitation are progressively altering the ecological habits and geographical distribution of pests and host plants in forest ecosystems (DeLucia et al. 2012; Skendžić et al. 2021). This may indirectly result in alterations to the structure and composition of forests, which could have a detrimental impact on the biodiversity of ecosystems (Coomes et al. 2014). The loss of biodiversity may result in a reduction in the stability and resistance of ecosystems, thereby rendering them more susceptible to other environmental stresses, such as pests, fires, or extreme weather events (Schmid and Pfisterer 2002). The 6th assessment report of the Intergovernmental Panel on Climate Change (IPCC) indicates that the global average surface temperature of land and oceans has risen by 1.1°C over the past 100 years. Furthermore, the IPCC report projects that surface temperatures could increase by more than 3.3°C–5.7°C by the end of the century (Torres López et al. 2017). In order to mitigate the negative impacts of climate change on forest ecosystems, a series of integrated management measures are required. These include the protection and restoration of ecosystems, the optimisation of pest management strategies, and the reduction of greenhouse gas emissions (Benayas et al. 2009; Vanderklift et al. 2022). Concurrently, species distribution models (SDM) are utilized to simulate prospective species migration pathways, thereby facilitating a more profound comprehension of the consequences of climate change on pests and host plants and the formulation of more precise and efficacious coping strategies (Tikhonov et al. 2017). The objective of these measures is to enhance the resilience of forest ecosystems, decelerate the loss of biodiversity, safeguard the ecological environment, and establish a robust foundation for sustainable global ecological development.

Ecological niche models (ENMs) have emerged as a valuable tool for elucidating the influence of environmental variables on species distribution patterns. This is accomplished by quantifying the correlation between species occurrence and various environmental variables (Kumar et al. 2014; Santana et al. 2019). The basic principle is to utilize the relationship between known species distribution records and environmental variables to hypothesize the potential geographic range of the species across varying spatial and temporal contexts (Xian et al. 2024). Currently, the most frequently utilized ecological niche models include Bioclim, Climex, Domain, and GARP models (Tsoar et al. 2007). These models have been extensively utilized in the prediction of animal and plant habitats, prioritization of pest and disease control, and species‐prioritized conservation areas, among other applications (Ge et al. 2019; Serrano‐Notivoli, Longares, and Cámara 2022). The MaxEnt model is a machine learning software based on the principle of maximum entropy (Zhang et al. 2024). It is capable of handling complex interactions between predictor variables more effectively than other ENMs and is therefore more effective in predicting the distribution of species (Wang et al. 2023). In addition, the algorithm is recognized for its ability to make reliable predictions for species with limited distribution ranges or small sample sizes compared to other ENMs, which gives its MaxEnt model a stronger advantage in terms of prediction accuracy and stability (Ouyang et al. 2022).

*Salix* is mainly distributed in the temperate regions of the Northern Hemisphere, with a low abundance or scattered distribution in South America and southern Africa, while there is no record of natural distribution in Oceania (Wu et al. 2015). China is one of the primary global distribution areas for the *Salix*, boasting a wealth of germplasm resources. Its rapid growth, short maturation period, diverse applications, and ease of propagation have contributed to its prominence as an essential afforestation and greening plant in China (Percy et al. 2014). It plays a pivotal role in the construction of an ecological environment and the advancement of the economy. Willow is a highly suitable raw material for paper manufacturing due to its exceptional fiber characteristics (Liao et al. 2019). At the same time, willow has a high biomass, is a high‐quality charcoal wood, and has the potential to be an important bioenergy tree species (Ran et al. 2022). From an ecological perspective, the role of willows should not be ignored; they can effectively control soil erosion, wind, sand, and improve saline soil (Ren et al. 2022). Furthermore, willows have the capacity to remove pollutants from the environment, combat air pollution, and reduce the greenhouse effect by absorbing carbon dioxide (Farag et al. 2024).

Willow trees are susceptible to a multitude of factors during their growth cycle, including impacts by pests and diseases, human interference, and other environmental factors (Larsen, Ucisik, and Trapp 2005; Simon et al. 2021; Zhang, Wang, and Lei 2022). In recent years, they have been particularly vulnerable to the damage caused by the trunk‐boring pest, *Anoplophora glabripennis* (Motschulsky 1854), an insect belonging to the genus *Anoplophora* of the family Cerambycidae in the order Coleoptera. It is widely distributed throughout China, including the provinces of Beijing, Tianjin, Hebei, Shanxi, Inner Mongolia, Liaoning, Jilin, Heilongjiang, Shanghai, Jiangsu, Zhejiang, Anhui, Fujian, Hubei, Hunan, Guangdong, Guangxi, Sichuan, and Yunnan (Wickham, Xu, and Teale 2012; Qin et al. 2024), it has also been reported in the USA, Canada, Austria, Germany, and even France (Bingjun et al. 2023). The insect primarily infests trees such as *Salix babylonica* Linn. and *Salix matsudana* Koidz, which represents one of the most significant pests of willow (Golec et al. 2018), causing major damage to the forest belt of the Three North Protective Forest in China. It has been classified as an important hazardous pest worldwide (Scully et al. 2014; Wang et al. 2020). The insect primarily infests the bast and xylem with larvae, resulting in the death of the tree in its entirety and a reduction in its lifespan (Heinrich and Collins 2017). Furthermore, the presence of numerous galleries can lead to a loss of economic value in the wood (Nordman et al. 2005; Li et al. 2020). Therefore, effective management of trunk‐boring pests is essential to ensure healthy forest growth and to restore the economic value of local forest.

The present study aimed to examine the potential suitable distribution areas and the major climatic factors influencing the trunk‐boring pest ( *A. glabripennis* ) and its host plants ( *S. babylonica* and *S. matsudana* ). The main objectives of the study include: (1) to identify the key bioclimatic variables affecting the distribution of *A. glabripennis* , *S. babylonica,* and *S. matsudana* in China; (2) to compare the differences in habitat distribution and area of the three species under the current period and future climate scenarios; and (3) to analyze the spatial evolution patterns of the three species and their future development trends. It is anticipated that this study will not only enhance our comprehension of the repercussions of climate change on the distribution of species but will also furnish a pivotal scientific foundation for strategic planning for ecological conservation and pest management in China. This may contribute to the well‐being of the ecosystem and the sustained prosperity of biodiversity.

## 2 Materials and Methods

### 2.1 Data on the Geographical Distribution of Species

**FIGURE 1:**
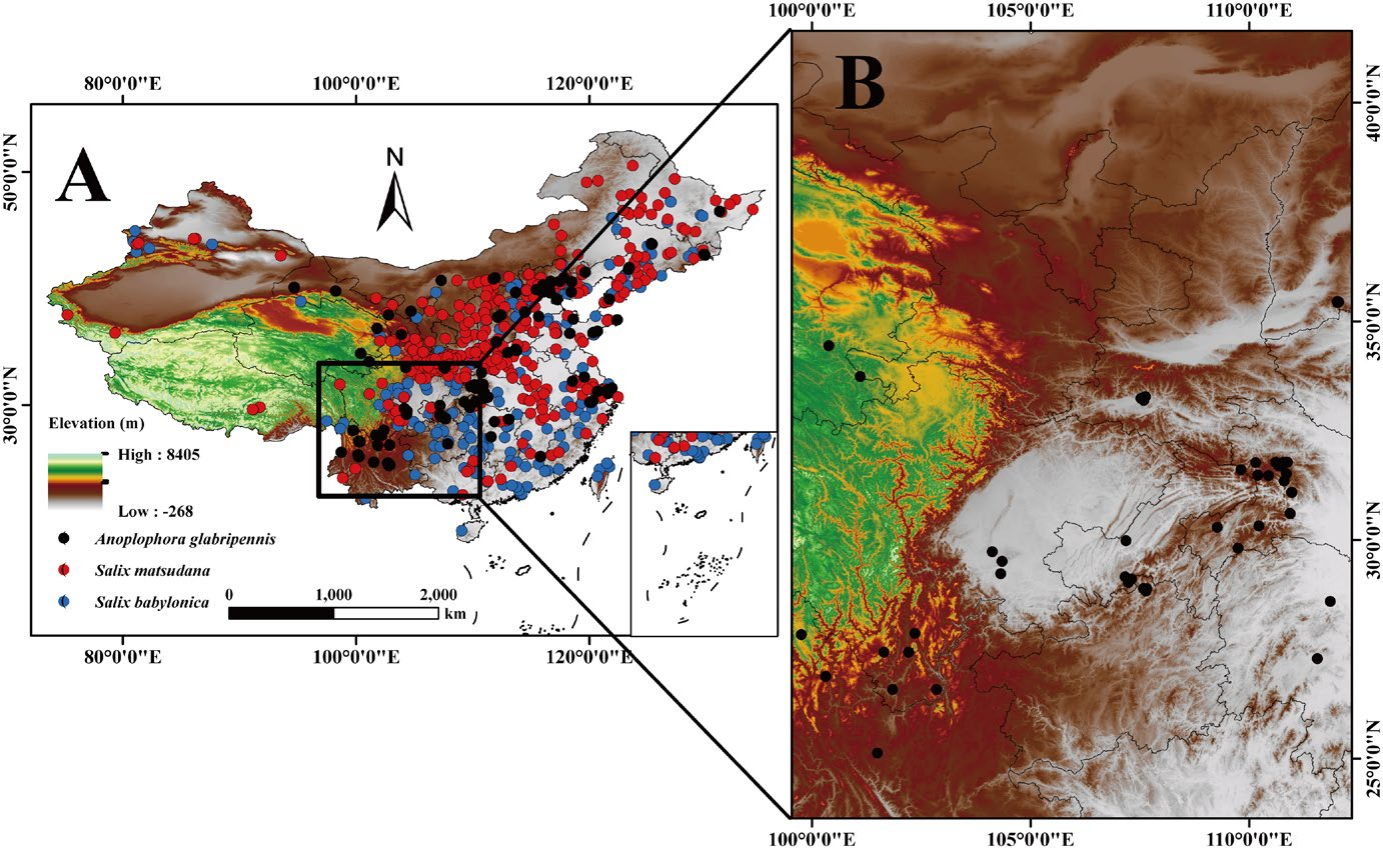
Occurrence data. (A) Records of the occurrence of *A. glabripennis* , and its host plants, *S. babylonica* and *S. matsudana*, within China; (B) field collection of *A. glabripennis* occurrences.

To generate the occurrence records of *S. babylonica* , *S. matsudana* , and *A. glabripennis* used in the modeling, we collected data from various sources (Figure 1A), including (1) book materials and online references (CNKI, https://www.cnki.net/; WOS, https://www.web of science.com/wos; NPSRC, http://www.cvh.ac.cn). (2) two online public databases, Global Biodiversity Information Facility (GBIF) (https://www.gbif.org/, were accessed on May 18, 2024; *S. babylonica* , https://doi.org/10.15468/dl.tv6bm7; *S. matsudana* , https://doi.org/10.15468/dl.xphep8; *A. glabripennis* , https://doi.org/10.15468/dl.d49psz) and iNaturalist (https://www.inaturalist.org/, were accessed on May 19, 2024). Additionally, (3) occurrences records of *A. glabripennis* were obtained from laboratory field surveys conducted from 2013 to the present year (Figure 1B). For occurrence points lacking specific latitude and longitude coordinates, Online Google Earth (http://ditu.google.cn/) was used to obtain this information. A total of 929 distribution records were obtained for *S. babylonica* , 1067 for *S. matsudana* , and 163 for *A. glabripennis* .

We used the “ENMTools” package (version 1.0.4) on the R platform(https://www.r‐project.org/, accessed October 1, 2023) remove duplicate data caused by overfitting due to spatial clustering (Warren et al. 2021). Finally, 336 points were retained for *S. babylonica* , 316 for *S. matsudana* , and 140 for *A. glabripennis* event records were retained for the construction of the MaxEnt model.

### 2.2 Environmental Data

The climate data were downloaded from WorldClim version 2.1 (https://www.worldclim.org/, accessed on January 24, 2021). The current period (considering 1970–2000 data as the current data set) and future climate data (2050s and 2070s) included 19 bioclimatic variables at a resolution of 2.5 arc minutes. For future climate data, we used the BCC‐CSM2‐MR global circulation model under four shared socioeconomic pathways (SSP: SSP1‐2.6, SSP2‐4.5, SSP3‐7.0, and SSP5‐8.5), which simulate the evolution of multi‐year temperature and precipitation data in China (Cerasoli, D'Alessandro, and Biondi 2022).

**FIGURE 2:**
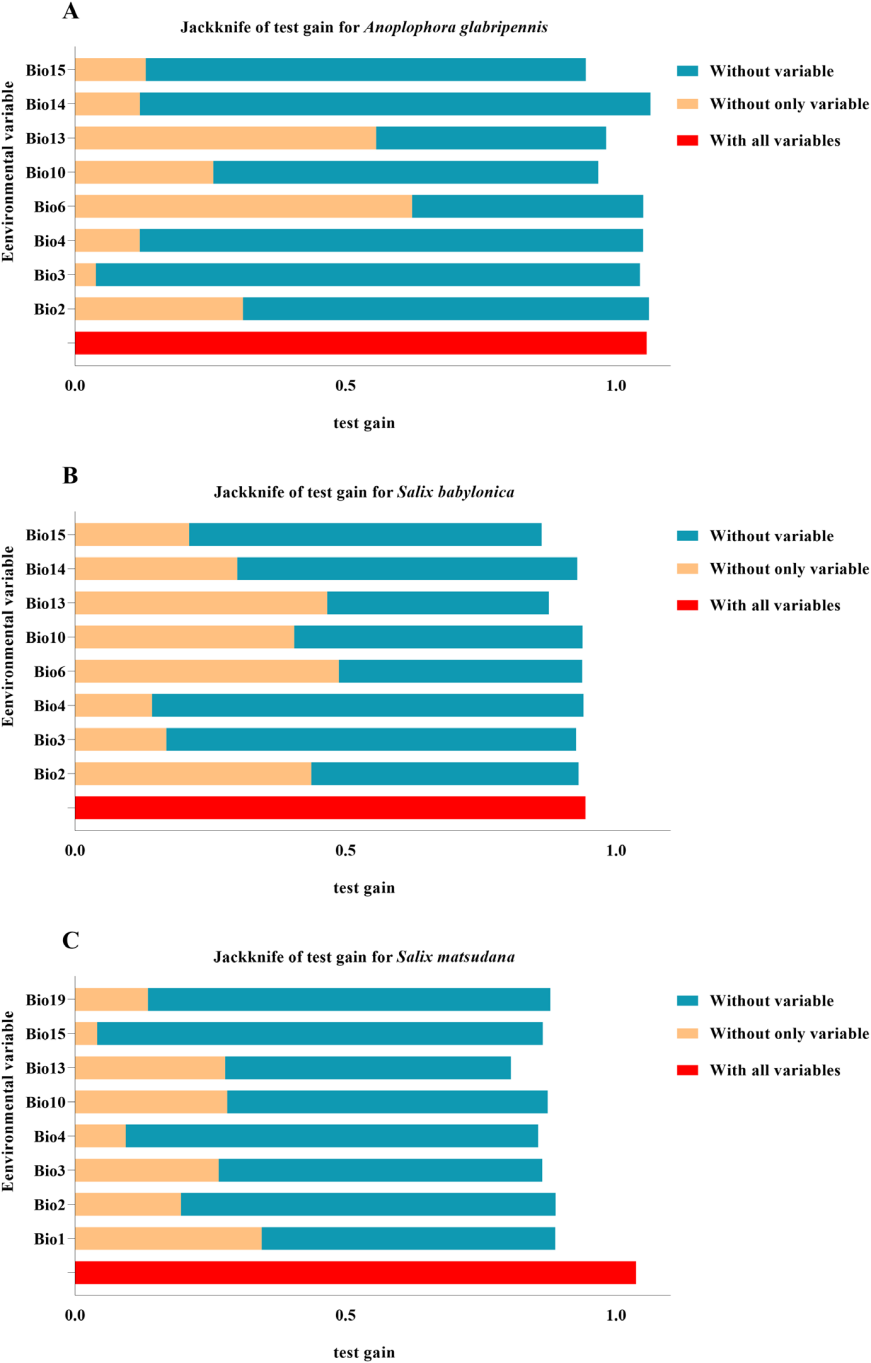
Significance of environmental variables was calculated using the Jackknife method. (A) *A. glabripennis* ; (B) *S. babylonica* ; and (C) *S. matsudana* .

There is a correlation between environmental variables, in order to avoid affecting the accuracy of model prediction, environmental variables can be used for model prediction only after correlation analysis (Evans and Jacquemyn 2022; Yoon et al. 2023). Firstly, the contribution rates of 19 environmental variables were obtained in the MaxEnt model using the Jackknife method, then the pearson correlation analysis was performed on the 19 environmental variables using the “ENMTools” package in the R software, and When the correlation coefficient between two environmental variables |*r*| > 0.9, the variable with the larger contribution is selected as the model input. This improves the ability of the model to accurately predict suitable habitats and reduces the risk of multicollinearity affecting model performance. Finally, 8 bioclimatic factors were retained to be imported into the MaxEnt model (Figure 2; Table 1).

**TABLE 1:** Correlation analysis and screening of environmental variables for *A. glabripennis* , *S. babylonica* , and *S. matsudana* .

Abbreviation	Environmental variables	Operation		
		*Anoplophora glabripennis*	*Salix babylonica*	*Salix matsudana*
Bio1	Annual mean temperature (°C)	Eliminate	Eliminate	Eliminate
Bio2	Mean diurnal range (°C)	Retain	Retain	Retain
Bio3	Isothermality	Retain	Retain	Retain
Bio4	Temperature seasonality	Retain	Eliminate	Retain
Bio5	Maximum temp of warmest month (°C)	Eliminate	Eliminate	Retain
Bio6	Minimum temp of coldest month (°C)	Retain	Retain	Retain
Bio7	Temperature annual range (°C)	Eliminate	Retain	Eliminate
Bio8	Mean temp of wettest quarter (°C)	Retain	Retain	Eliminate
Bio9	Mean temp of driest quarter (°C)	Eliminate	Eliminate	Eliminate
Bio10	Mean temp of warmest quarter (°C)	Eliminate	Eliminate	Eliminate
Bio11	Mean temp of coldest quarter (°C)	Eliminate	Eliminate	Eliminate
Bio12	Annual precipitation (mm)	Eliminate	Retain	Retain
Bio13	Precipitation of wettest month (mm)	Eliminate	Eliminate	Eliminate
Bio14	Precipitation of driest month (mm)	Retain	Retain	Retain
Bio15	Precipitation seasonality (mm)	Retain	Retain	Retain
Bio16	Precipitation of wettest quarter (mm)	Retain	Eliminate	Eliminate
Bio17	Precipitation of driest quarter (mm)	Eliminate	Eliminate	Eliminate
Bio18	Precipitation of warmest quarter (mm)	Eliminate	Eliminate	Eliminate
Bio19	Precipitation of coldest quarter (mm)	Eliminate	Eliminate	Eliminate

### 2.3 Model Construction and Evaluation

Regularized multipliers (RMs) and feature combinations (FCs) are key parameters in the MaxEnt model, and optimizing these parameters can significantly improve the accuracy of the model (Fang et al. 2022). The FCs consist of five feature types: L (linear), Q (quadratic), H (hinge), P (product), and T (threshold). The parameters of the RMs and FCs were tuned using the “ENMeval” package in the R software. Initially, the RMs were set from 0 to 4 with an interval of 0.5, depending on the retained distribution data and environmental variables.To determine the optimal parameter combinations, six FCs were specified: L, LQ, H, LQH, LQHP, and LQHPT (Miao et al. 2024). Subsequently, 48 models were constructed containing different combinations of the RMs and FCs, and the model with the smallest delta was produced. Additional parameters for the optimal model were set as follows: 25% of the distribution points of each species were selected as the test set and 75% as the training set. The maximum number of iterations was set to 5000, and the maximum number of background points was limited to 10,000, repeated 10 times (Fernandez Diaz, Mendez Martinez, and Fernandez Suarez 2014). The closer the test omission rate is to the theoretical omission rate, the more accurate the model construction is. The significance of each variable was measured using the product Jackknife method, and response curves were generated.

The receiver operating characteristic (ROC) curve and area under curve (AUC) value can be effectively used to evaluate the accuracy and effectiveness of the model. The AUC ranges from 0 to 1, with higher values indicating better model performance (Campos et al. 2023). A model with an AUC value less than 0.5 indicates poor predictive performance. Models with AUC values between 0.7 and 0.9 are considered to have good predictive validity. Models with AUC values greater than 0.9 are considered excellent, indicating high accuracy in prediction (Zhang et al. 2021).

### 2.4 Changes in the Potential Distribution Areas of *A. glabripennis* and Its Host Plants

In this study, the average value after 10 repetitions of the optimal MaxEnt model was used as the final result, based on the logical value of the probability of existence (*p*) of the species. The final result was converted into raster form and visualized using ArcGIS Map software. *S. babylonica* , *S. matsudana,* and *A. glabripennis* suitability classes were classified into four classes according to the United Nations Intergovernmental Panel on Climate Change (IPCC) report and were divided into four classes, namely unsuitable habitat (*p* < 0.05), low suitable habitat (0.05 ≤ *p* < 0.33), medium suitable habitat (0.33 ≤ *p* < 0.66), and highly suitable habitat (0.66 ≤ *p* ≤ 1) (Liu et al. 2022; Gao et al. 2023). The number of rasters in each class was counted, and the proportion of the area of each class of suitable habitat was calculated, and graphs were made using GraphPad Prism 9.0 software.

### 2.5 Dynamics of *A. glabripennis* and Its Host Plants under Future Climate Scenarios

The “Distribution Changes between Species Distribution Models (SDMs)” function in the “SDMToolbox v2.6 toolbox” in ArcGIS Map (Brown and Anderson 2014) was used to investigate the dynamic changes of *A. glabripennis* and its host plants, *S. babylonica and* *S. matsudana* , under future climate scenarios (Zhang et al. 2024a). As a result, four outputs were obtained: “Expansion”, “No occupancy”, “Unchanged”, and “Contraction”.

### 2.6 Changes of Spatial Pattern of *A. glabripennis* and Its Host Plants under Future Climate Scenarios

The potential distribution centers of the areas of *A. glabripennis* and its host plants, *S. babylonica* and *S. matsudana* , with a probability (*p*) of existence greater than 0.05, were calculated for each future period using the “Centroid Changes (Lines)” tool in SDMToolbox v2.6 (Brown and Anderson 2014). The locations where the potential distribution centers shifted were then compared for different scenarios in the current and future periods (including four different carbon emission scenarios). Line segments were used to connect the calculated potential distribution center points, creating shift routes that reflect the spatial shift routes of the main suitable areas of *A. glabripennis* and its host plants under future climate conditions.

## 3 Result

### 3.1 Optimization of the Model and Its Accuracy

**FIGURE 3:**
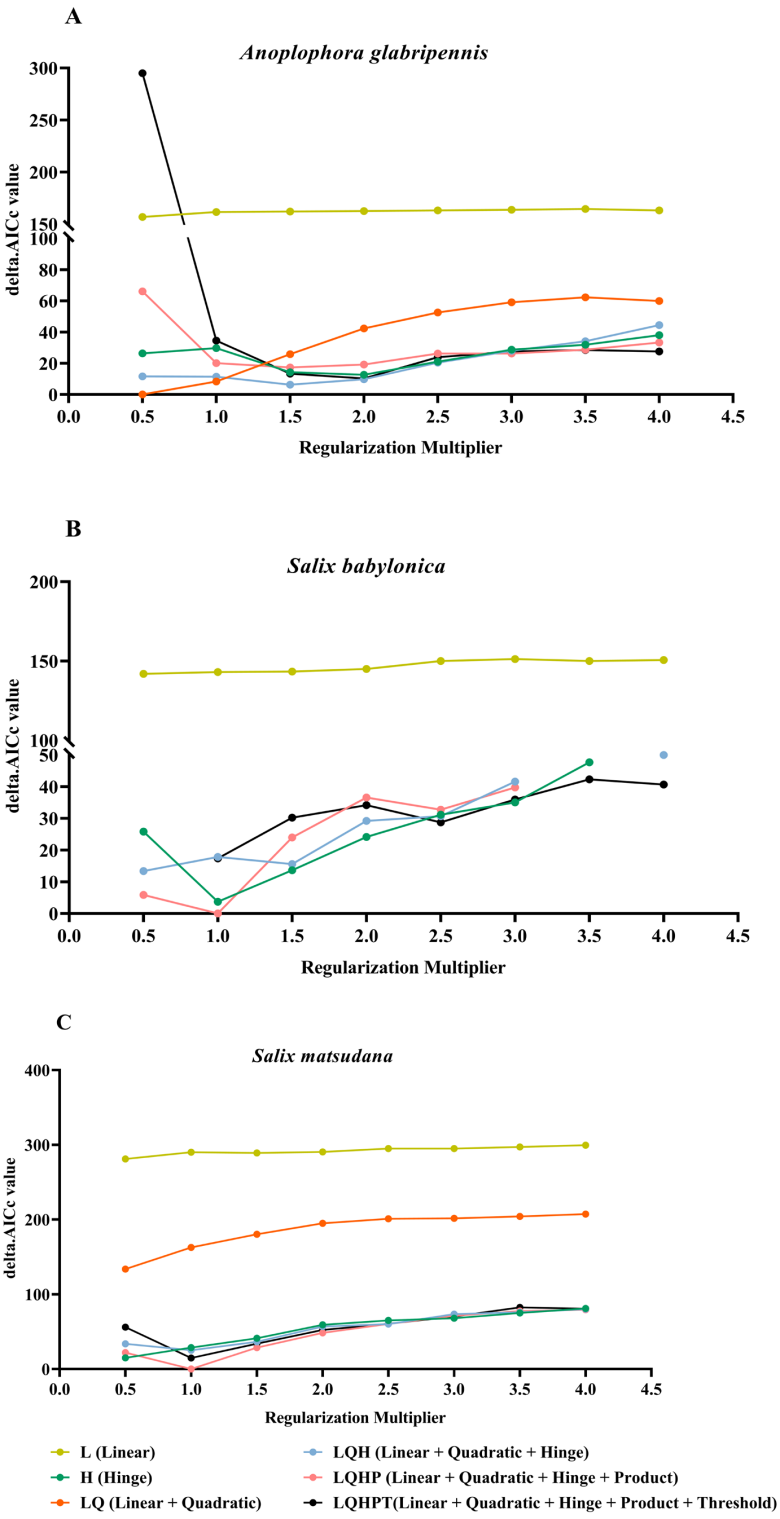
Delta. AICc for *A. glabripennis* , and its host plants, *S. babylonica* and *S. matsudana* from models under different parameter combinations. (A) A. glabripennis, (B) S. babylonica, (C) S. matsudana.

The simulated predictions were evaluated by optimizing the average AUC value and TSS value of the 10 outcomes in the MaxEnt model (Table 2). The optimized model achieved the delta.AICc value of 0, which is the most likely to be the optimal model. For the *S. babylonica* and *S. matsudana* optimization model, the smallest delta.AICc value was obtained with RMs = 1 and an FCs of LQHP. In the case of the *A. glabripennis* optimization model, the smallest delta.AICc value was achieved with RMs = 0.5 and an FCs of LQ. The average AUC and TSS values for the three species exceeded 0.8 (Table 2). This outcome suggests that the model's level of fit is highly accurate, and it can effectively simulate the suitable habitat distribution for *S. babylonica* , *S. matsudana*, and *A. glabripennis* (Figure 3).

**TABLE 2:** Model accuracy evaluation for *A. glabripennis* , *S. babylonica* , and *S. matsudana* .

Scenario	*Anoplophora glabripennis* (Average)			*Salix babylonica* (Average)			*Salix matsudana* (Average)		
	Train AUC	Test AUC	TSS	Train AUC	Test AUC	TSS	Train AUC	Test AUC	TSS
Current	0.9699	0.9410	0.8951	0.9005	0.8764	0.8845	0.9522	0.9374	0.8852
Future‐SSP1‐2.62040–2060	0.9668	0.9398	0.8820	0.8951	0.8726	0.8612	0.9485	0.9334	0.8764
Future‐SSP1‐2.62060–2080	0.9633	0.9394	0.8715	0.8996	0.8779	0.8741	0.9498	0.9348	0.8656
Future‐SSP2‐4.52040–2060	0.9644	0.9413	0.8645	0.8922	0.8688	0.8523	0.9514	0.9356	0.8613
Future‐SSP2‐4.52060–2080	0.9614	0.9343	0.8813	0.8954	0.8709	0.8450	0.9528	0.9384	0.8514
Future‐SSP3‐7.02040–2060	0.9629	0.9354	0.8541	0.8981	0.8772	0.8455	0.9513	0.9424	0.8874
Future‐SSP3‐7.02060–2080	0.9636	0.9406	0.8668	0.8950	0.8706	0.8660	0.9491	0.9348	0.8762
Future‐SSP5‐8.52040–2060	0.9616	0.9363	0.8465	0.8967	0.8786	0.8561	0.9523	0.9337	0.8431
Future‐SSP5‐8.52060–2080	0.9628	0.9355	0.8893	0.8936	0.8754	0.8304	0.9515	0.9420	0.8840

### 3.2 Important Environmental Variables

**FIGURE 4:**
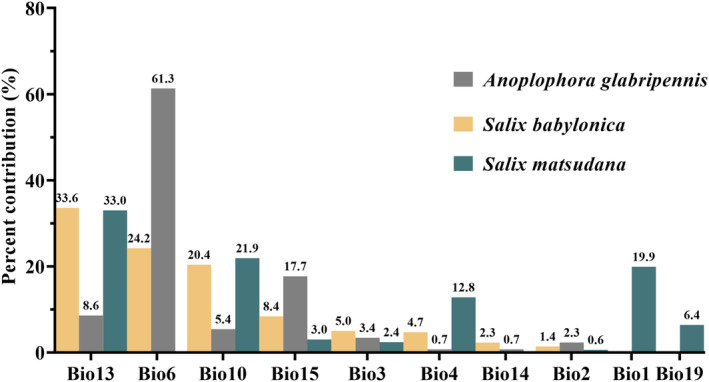
The contribution rates of the environmental variables in MaxEnt model.

The analysis of single‐factor contributions indicated that precipitation of the wettest month (Bio13, 33.6%), minimum temp of the coldest month (Bio6, 24.2%), mean temp of the warmest quarter (Bio10, 20.4%), precipitation seasonality (Bio15, 8.4%), and isothermality (Bio3, 5%) made significant contributions to the prediction model for *S. babylonica* ; the cumulative contribution value was reached up to 91.6%. Among the *S. matsudana* environmental variables selected, the important factors were precipitation of the wettest month (Bio13, 33%), followed by mean temp of warmest quarter (Bio10, 21.9%), annual mean temperature (Bio1 19.9%), temperature seasonality (Bio4, 12.8%), and precipitation of coldest quarter (Bio19, 6.4%), the cumulative contribution rate was 94%. Minimum temp of the coldest month (Bio6, 61.3%), precipitation seasonalit (Bio15, 17.7%), precipitation of the wettest month (Bio13, 8.6%), and mean temp of warmest quarter (Bio10, 5.4%); these four factors were the strongest predictors of *A. glabripennis* occurrence with a contribution of 93% (Figure 4).

**FIGURE 5:**
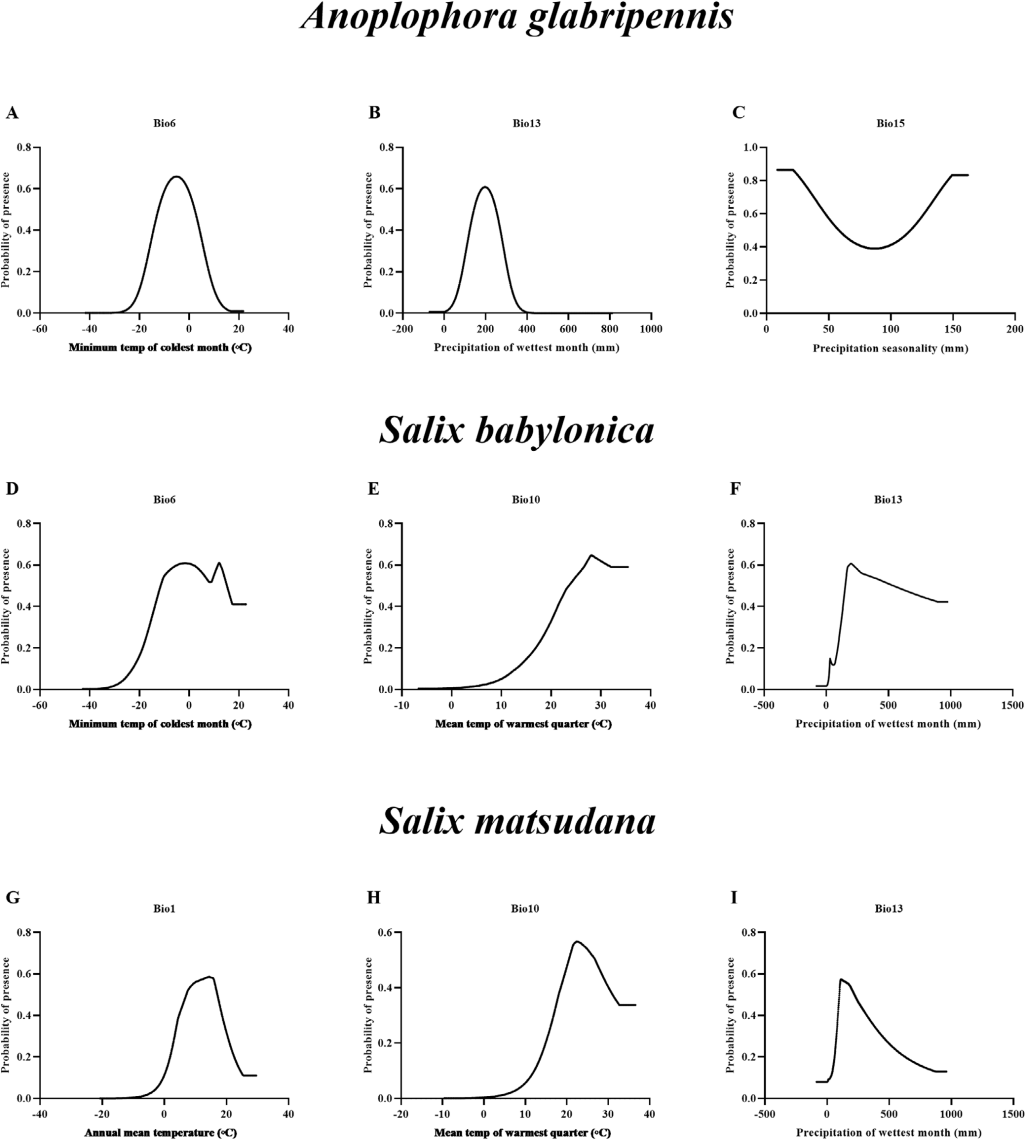
Response curves for bioclimatic factors. (A) Bio6; (B) Bio13; (C) Bio15; (D) Bio6; (E) Bio10; (F) Bio13; (G) Bio1; (H) Bio10; and (I) Bio13.

In addition, the response variable curves showed that the suitability of *A. glabripennis* in relation to Bio6 and Bio13 exhibited a normal distribution, specifically, a gradual increase in suitability as the bioclimatic variables increased to the apex and then a gradual decrease. However, the suitability of *A. glabripennis* with Bio15 showed a decrease followed by a gradual increase. The probability of survival of host plants ( *S. babylonica* and *S. matsudana* ) increased with increasing Bio10 and Bio13, and after reaching the optimum interval, the suitability of *S. babylonica* and *S. matsudana* gradually decreased (Figure 5).

### 3.3 Potential Geographical Distribution Under Current Climate Conditions

**FIGURE 6:**
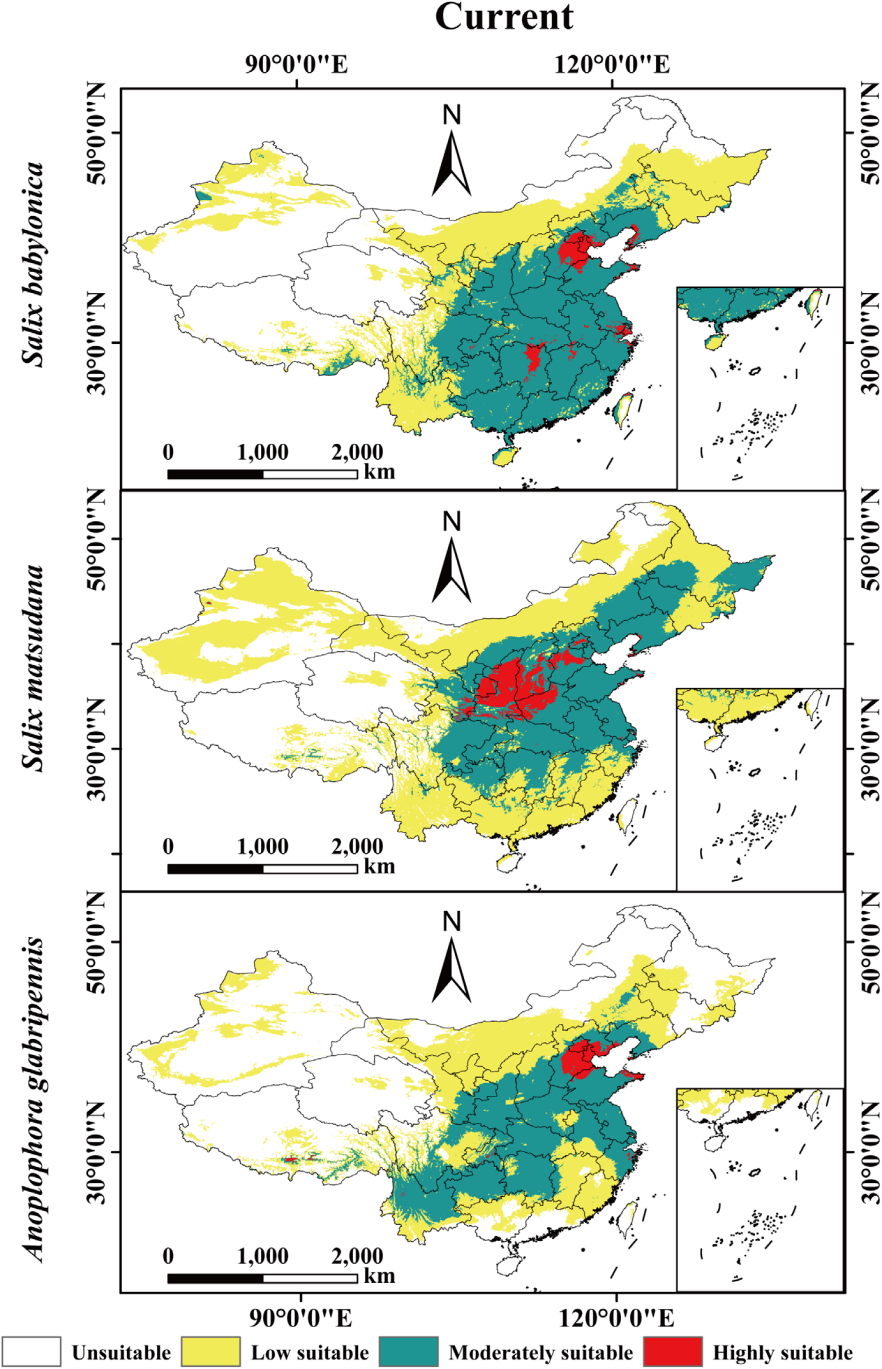
Distribution of suitable habitat for *A. glabripennis* , and its host plants, *S. babylonica* and *S. matsudana* in China under current climate conditions.

The MaxEnt projections indicate the geographic distribution of pest and host plants under the current climate scenario, as depicted in Figure 6. The potential suitable habitat ranges of the three species were consistent with the range of established distribution areas, indicating that the model developed in this study was effective and accurate in simulating the potential suitable habitat ranges of pests and host plants.

Under the current climatic conditions, the total area of suitable habitat for the two host plants ( *S. babylonica* and *S. matsudana* ) was 559.01 × 10^4^ km^2^ and 660.44 × 10^4^ km^2^, respectively. The area of highly suitable habitat for *S. babylonica* was 16.47 × 10^4^ km^2^, which accounted for 1.71% of the total area of China. The area of moderately suitable habitat was 247.93 × 10^4^ km^2^, accounting for 28.59% of the total area of China. The area of low suitable habitat was 267.61 × 10^4^ km^2^, accounting for 27.82% of the total area of China (Figure S1; Table S1). The area of highly suitable habitat of *S. matsudana* was 32.47 × 10^4^ km^2^, accounting for 3.38% of the total area of China. The area of moderately suitable habitat was 241.26 × 10^4^ km^2^, accounting for 25.09% of the total area of China. The area of low suitable habitat was 386.71 × 10^4^ km^2^, accounting for 40.21% of the total area of China (Figure S1; Table S1). However, the total area of suitable habitat for *A. glabripennis* was 489.33 × 10^4^ km^2^. Among them, the area of highly suitable habitat was 12.74 × 10^4^ km^2^, accounting for 1.32% of the total area of China. The area of moderately suitable habitat was 197.13 × 10^4^ km^2^, accounting for 20.50% of the total area of China. The area of low suitable habitat was 279.46 × 10^4^ km^2^, accounting for 29.06% of the total area of China (Figure S1; Table S1).

### 3.4 Potential Geographical Distribution Under Future Climate Scenarios

**FIGURE 7:**
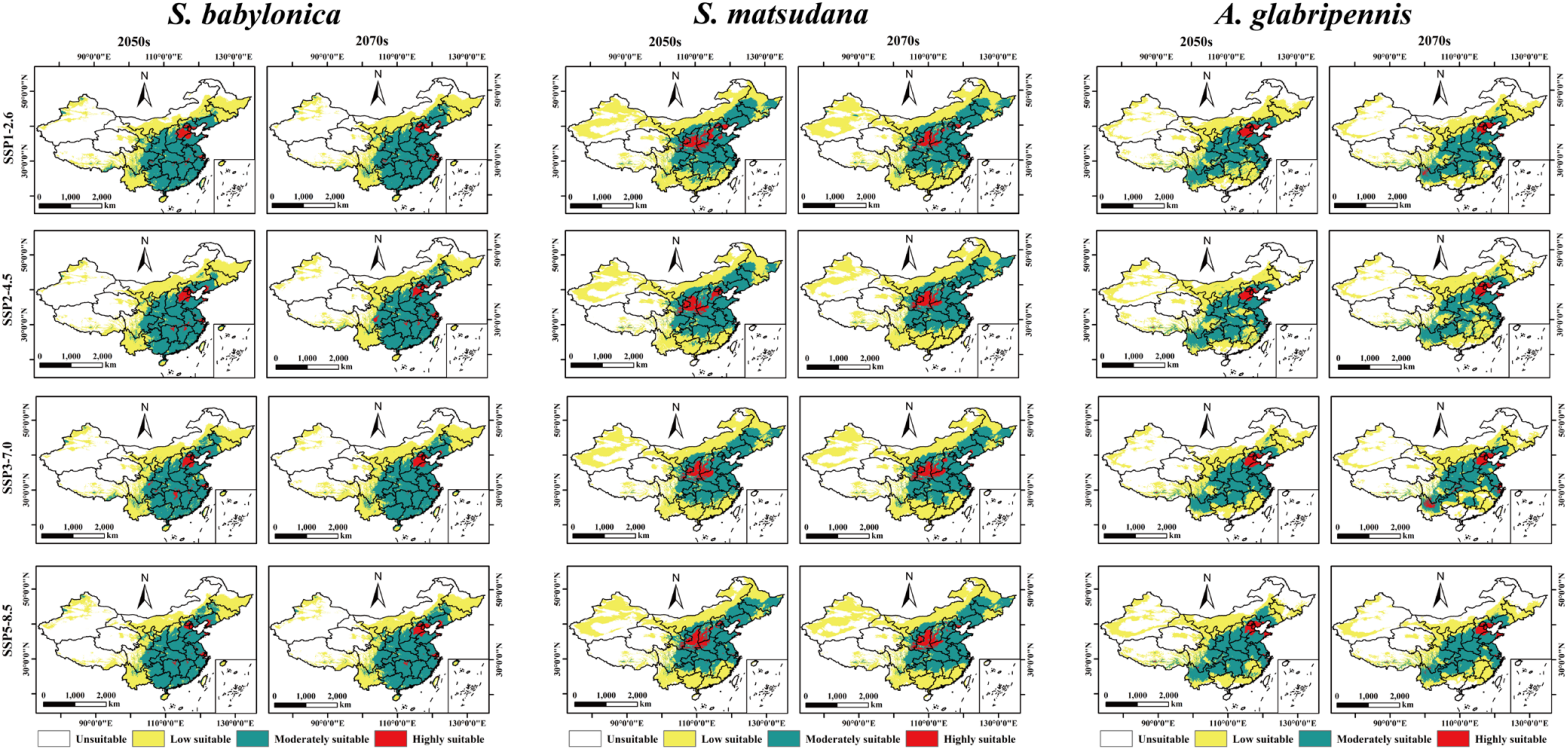
Potential suitable distribution habitat for *A. glabripennis* , and its host plants, *S. babylonica* and *S. matsudana* in China under future climate scenarios.

Under different future climate scenarios, the range of suitable habitats for *A. glabripennis* and its host plants ( *S. babylonica* and *S. matsudana* ) shows relatively little change compared to the current climate scenario (Figure 7). The suitable areas of *S. babylonica* in the future are all smaller than that in the current period, and the suitable habitat area of *S. babylonica* is 541.19 to 555.36 × 10^4^ km^2^, accounting for 56.27%–57.74% of the total area of China. Among them, under the SSP3‐7.0 scenario in the 2070s, *S. babylonica* has the largest area of suitable habitat, and under the SSP2‐4.5 scenario in 2070s has the smallest area of suitable habitat. Analysis of suitable area under the four future climate scenarios showed that SSP1‐2.5, SSP2‐4.5, and SSP5‐8.5 show a continuous trend of decreasing area of *S. babylonica* suitable habitat over time compared to current climate. However, the SSP3‐7.0 scenario showed a decreasing and then increasing trend in suitable habitat area (Figure S1; Table S1).

The potential suitable habitat area of *S. matsudana* ranges from 640.81 to 677.08 × 10^4^ km^2^, accounting for 66.63%–69.82% of the total area of China, and the suitable area under any future scenario has a temporal dependence, which is characterized by a continuous increase in the suitable area with the increase of time (Figure S1; Table S1). The area of potentially suitable habitat for *A. glabripennis* ranges from 470.43 to 572.77 × 10^4^ km^2^, accounting for 48.94%–59.55% of the total area of China. Under the SSP3‐7.0 scenario in the 2070s, the area of potentially suitable area is smaller than the current period, except for the area of suitable area under any other future scenario, which is larger than the current period. In addition, the suitable area of *A. glabripennis* under any future scenario shows an increase and then a decrease over time.

### 3.5 Centroid Shift

**FIGURE 8:**
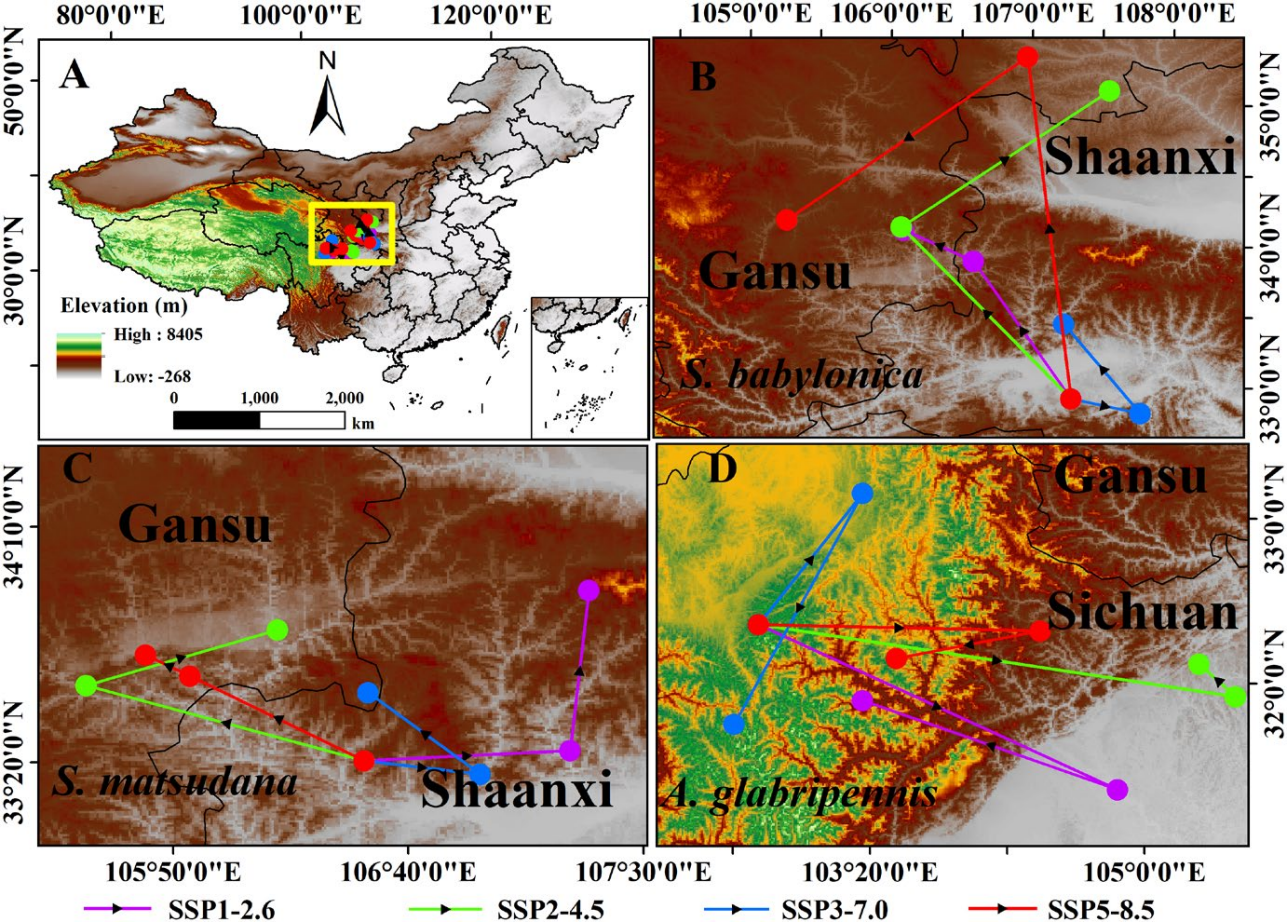
Spatial changes in the geometric centre of potential distribution areas by 2050 and 2070, *A. glabripennis* , and its host plants, *S. babylonica* and *S. matsudana*, under two different scenarios of a changing climate. (A) Geometric centers of distribution of the three species within China, (B) A. glabripennis, (C) S. babylonica, (D) S. matsudana.

Under different climate scenarios, the center of the three species habitat exhibits varying shifts. Under the current climate scenario, the distribution centers of *S. babylonica* (32.91° N, 107.27° E) and *S. matsudana* (33.34° N, 106.51° E) habitats are located in Shaanxi province, while the center of distribution of *A. glabripennis* (32.36° N, 102.66° E) is located in Sichuan province (Figure 8; Table S2).

In all future climate scenarios, the potential distribution center of *A. glabripennis* is located in Sichuan Province. In the SSP1‐2.6 path, the distribution center of *A. glabripennis* was located at 31.36° N, 104.84° E in the 2050s and shifted by 78.67 km to the southeast and was located at 31.90° N, 103.29° E in the 2070s. In the SSP2‐4.5 climate scenario, by the 2050s, the center moves southeast to 31.92° N, 105.56° E, and northwest by 277.76 km to 32.12° N, 105.33° E by the 2070s. In the SSP3‐7.0 pathway, the distribution center of *A. glabripennis* was located at 33.15° N, 103.29° E in the 2050s and at 31.75° N, 102.51° E in the 2070s, shifting 68.47 km to the north. In the SSP5‐8.5 climate scenario, by the 2050s, the center moves east to 32.32° N, 104.37° E, and southwest by 82.15 km to 32.15° N, 103.49° E by the 2070s (Figure 8, Table S2).

In the SSP1‐2.6 path, the distribution centers of *S. babylonica* and *S. matsudana* were located at 33.90° N, 106.58° E (in Shaanxi) and 33.37° N, 107.24° E (in Shaanxi), respectively, by the 2050s, and shifted by 172.46 km to the northwest and 99.62 km to the northeast by the 2070s, to be located at 34.12° N, 106.08° E (in Gansu) and 33.94° N, 107.31° E (in Shaanxi), respectively. In the SSP2‐4.5 pathway, the distribution center of *S. babylonica* in the 2050s is located at 34.15° N, 106.07° E (in Gansu), and the distribution center in the 2070s is located at 35.11° N, 107.54° E (in Gansu), with the distribution center shifting 243.40 km to the north. The distribution center of *S. matsudana* was also located at 33.61° N, 105.53° E (in Shaanxi) in the 2050s and at 33.80° N, 106.20° E (in Shaanxi) in the 2070s, shifting 58.56 km to the northwest. In the SSP3‐7.0 pathway, the distribution center of *S. babylonica* was located at 32.82° N, 107.76° E (in Shaanxi) in the 2050s and at 33.46° N, 107.22° E (in Shaanxi) in the 2070s, shifting 59.23 km to the north, and the distribution center of *S. matsudana* was also located at 33.29° N, 106.92° E (in Shaanxi) in the 2050s and at 33.58° N, 106.53° E (in Gansu) in the 2070s, shifting 26.78 km to the north. In the SSP5‐8.5 path, the distribution centers of *S. babylonica* and *S. matsudana* were located at 35.35° N, 106.96° E (in Gansu) and 33.64° N, 105.89° E (in Gansu), respectively, by the 2050s, and shifted by 233.97 km and 83.03 km to the northwest by the 2070s, to be located at 34.20° N, 105.26° E (in Gansu) and 33.71° N, 105.74° E (in Gansu), respectively (Figure 8; Table S2).

### 3.6 Relative Changes in Potential Distribution Under Future Climate Scenarios

**FIGURE 9:**
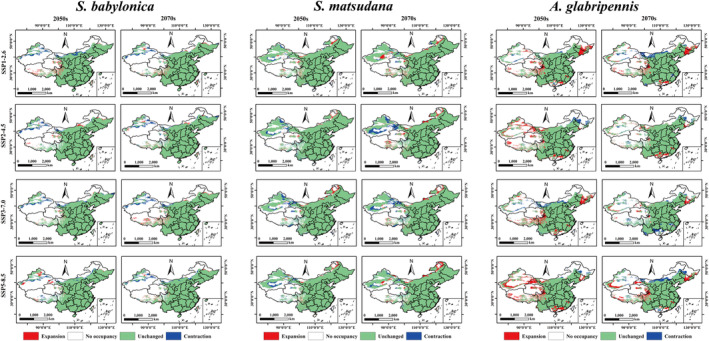
The changes in the distribution of suitable habitats of *A. glabripennis* , and its host plants, *S. babylonica* and *S. matsudana*, under different climate scenarios.

Relative changes in the potential distribution of pests and host plants were derived by analyzing the differences in the current and future changes in the size of suitable distribution areas (Figure 9). The results indicated that the suitable distribution area of *A. glabripennis* would change under future climate scenarios, suggesting that the suitable habitat for *A. glabripennis* would increase, with expansion areas ranging from 20.63 to 90.47 × 10^4^ km^2^. Expansion would mainly occur in Heilongjiang, Jilin, Liaoning, Xinjiang, Qinghai, Gansu, Guangdong, Guangxi, Fujian, Sichuan, Yunnan, and Xizang, and declined would mainly occur in Gansu, Xinjiang and Guangxi. Among them, the expansion area is the largest in the SSP5‐8.5‐2050s, while the declined area is the largest in the SSP5‐8.5 scenario in 2070s (Figure S2; Table S3). However, the potentially suitable distribution areas of the host plants *S. babylonica* and *S. matsudana* will declined further. The *S. babylonica* declineded over an area of 13.87 to 40.89 × 10^4^ km^2^, with the declined area mainly in Inner Mongolia, Gansu, Xinjiang, and Yunnan, and the expansion area in Inner Mongolia, Qinghai, Sichuan, and Xizang. Among them, SSP2‐4.5‐2070s has the largest declined area, and SSP1‐2.6‐2070s has the smallest declined area. *S. matsudana* has a declined area of 22.27 to 28.52 × 10^4^ km^2^, with the declined area mainly distributed in Inner Mongolia, Gansu, and Xinjiang, and the expansion area in Inner Mongolia, Xinjiang, Qinghai, Sichuan, and Xizang. Among them, the declined area of SSP2‐4.5‐2070s is the largest, and the declined area of SSP1‐2.6‐2070s is the smallest (Figure S2; Table S3).

## 4 Discussion

Ecologists face a number of challenges in predicting how organisms will respond to future climate change. These challenges arise not only from the inherent complexity and uncertainty of ecosystems themselves but also from the multitude of interactions that occur within them, including food chains, competitive relationships, and symbiotic relationships. These interactions may indirectly affect ecosystem dynamics (Liu et al. 2021; Pound et al. 2021). Consequently, ecologists must assess the adaptive capacity of different species and predict how they may migrate and adapt to new environmental conditions (Zucchini 2012; Zhao et al. 2024). In this study, we employed food chain logic and integrated it with the MaxEnt model to conduct a potential distribution area and movement route analysis of the trunk‐boring pest *A. glabripennis* and its host plants ( *S. babylonica* and *S. matsudana* ). The objectives of the study were to provide a scientific basis for the effective management of *A. glabripennis* under climate change conditions and to provide theoretical guidance for a deeper understanding of the adaptive management and sustainable utilization of its host plants ( *S. babylonica* and *S. matsudana* ).

### 4.1 The Reliability of the Simulation Results

The accuracy of a species distribution model is affected by factors such as input data (including species distribution data points and environmental variables) and the selection of the software's own parameters, which are directly related to the results of the model output (Franklin et al. 2009). In this study, the researchers initiated field surveys in 2013, amassing a more comprehensive array of distribution point data than that available in the literature and public online databases. The surveys were conducted in multiple provinces and cities in China, resulting in an increase of 54 sample distribution points for *A. glabripennis* , and a trend of westward expansion of its population was observed. In order to enhance the predictive accuracy of the model and prevent overfitting, the parameters of the MaxEnt model were optimally adjusted in this study. Following the adjustment of the parameters, all AUC values of the model exceeded 0.80, indicating high accuracy and reliability in predicting pests and host plants using the optimized MaxEnt model (Wang et al. 2024). This study was analyzed through field surveys and the adjustment of model parameters, which not only improved the accuracy of species habitat prediction but also provided an important scientific basis for species monitoring and ecological protection under future climate scenarios.

### 4.2 Analysis of the Main Bioclimatic Variables

Although the main climatic variables affecting *S. babylonica* and *S. matsudana* differ slightly, the vast majority of the dominant factors overlap with each other, which may be due to the fact that the study species are all from the genus *Salix* in the family Salicaceae, and the species are closely related to each other, which makes them have similar habitat requirements and ultimately subjects to the same bioclimatic factors (Rog et al. 2020). In this study, we found that the mean temp of the warmest quarter (Bio10) and precipitation of the wettest month (Bio13) were the dominant factors influencing the species and their potential distributions of Willow ( *S. babylonica* and *S. matsudana* ) in China through the combined analysis of contribution rate, sequence importance value and the Jackknife method, which proved that the combined effect of heat and moisture conditions plays an important role in shaping the species and their potential distribution patterns of willow in China. With climate change, there will be a gradual decrease in precipitation and a decline in soil water content in the future, thus exacerbating drought stress (Luce et al. 2016). The instability of precipitation and temperature may threaten the distribution of *S. babylonica* and *S. matsudana* , leading to changes in their ranges and thus altering their distribution patterns. The actual distribution area of *S. babylonica* and *S. matsudana* and the potential distribution area predicted by simulation both showed that the highly suitable growth area of *S. babylonica* and *S. matsudana* is mainly located in the temperate continental monsoon climate, which has a warm climate, low seasonal variation in temperature and moderate precipitation, which is consistent with the relationship between the distribution of *S. babylonica* and *S. matsudana* and bioclimatic factors found in this study. However, the probability of presence of *A. glabripennis* changed with climatic variables, and among the eight dominant environmental variables minimum temp of the coldest month (Bio6) and precipitation seasonality (Bio15) had the greatest influence on the distribution of *A. glabripennis* in the potential suitability areas. Similarly, Zhang, Wang, and Lei (2022). showed that changes in annual mean temperature (Bio1) and precipitation of the driest month (Bio14) had a crucial role in the potential distribution area of *A. glabripennis* .

### 4.3 Geographic Distribution of Suitable Habitats and Movements in Potential Range Centers for *A. glabripennis* and Its Host Plants, *S. babylonica* and *S. matsudana* , Under Climate Change

According to the projections of *S. babylonica* and *S. matsudana* , the increasing temperature and drought stress in the future will lead to the decline of the potential range area of the two willow species, and this decline phenomenon will gradually increase with the intensification of climate change. It is also found that with climate change, the distribution areas of *S. babylonica* and *S. matsudana* have a tendency to migrate northward, which indicates that climate change will affect the distribution of *S. babylonica* and *S. matsudana* to a certain extent, resulting in changes in the distribution area of the species as well as the scope of the distribution area (Davis et al. 2018). Similarly, changes in the size of a species' range indirectly reflect changes in climate. In addition, the premise of the species distribution model assumes that the ecological niche of the species will not change. Hence, in the projected future climate scenarios, they can only survive and be distributed in the same environment as the climate of their current ranges (Santini et al. 2021). This would result in species ranges migrating along the negative gradient of climate change in a warmer and drier future climate. Thus, this species distribution response to climate change is also a combination of hydrothermal synergies.

In our prediction results, we found that the range area of *A. glabripennis* showed a gradual expansion trend as the future climate tends to warm and dry up. This phenomenon may be attributed to the warming of the global climate leading to an increase in the effective cumulative temperature of insects, which in turn lengthens their growth cycle and creates favorable conditions for insect growth and development. However, there exists a threshold for the adaptability of insects to increased temperatures, and once this threshold is exceeded, warming will inhibit the growth of the species and lead to a gradual reduction in their distribution areas. In addition, while most species will face the challenge of decreasing range size under global warming, species *A. glabripennis* showed a trend of increasing range size in the prediction, which suggests that the species may have a strong adaptive capacity to adapt to the environment (Gong et al. 2023). Our study further reveals that not all climate change scenarios result in a northward expansion of *A. glabripennis* range. This may be because one of the key factors affecting the distribution of *A. glabripennis* is the variance of temperature seasonality (Bio4), a climatic variable whose future changes in latitude are not simply linear compared to other bioclimatic variables, which may result in the species range not simply shifting to higher latitudes under some projected scenarios (Powell and Logan 2005). On the other hand, climate change is driving *A. glabripennis* to spread not only to higher latitudes but also to expand to higher elevations. It is worth noting that the tendency to spread to higher altitudes may be more pronounced than the tendency to migrate to higher latitudes, which may mask clear signs of a northward shift in range (Zhang et al. 2024b).

### 4.4 Limitations of This Research

Our study used bioclimatic variables to predict the potential geographic distribution of trunk‐boring pests and their host plants in forest ecosystems with high prediction accuracy but with some limitations. First, there are many uncertainties in future climate change. In this study, only the BCC‐CMS2‐MR climate model was used to simulate the potential suitable areas for *A. glabripenni* and its host plants, *S. babylonica* and *S. matsudana* , in China, and future studies to predict their potential suitable habitats should use several global climate models (GCM). This will provide a wider range of options for decision‐makers to develop forest ecological conservation measures in practice. In addition, this study only considered the effects of bioclimatic variables among abiotic factors on species distribution, ignoring factors such as solar radiation, the normalized difference vegetation index, and topography. In fact, biotic factors such as competition, predation, and diseases of the species itself also affect species distribution.

Nevertheless, our results provide important insights into the distribution of trunk‐boring pests and their host plants in forest ecosystems. We emphasize that the aim of this study is to provide an initial predictive framework to guide the effective management of trunk‐boring pests and to provide a scientific basis for the conservation, introduction and promotion of *Salix* resources. Follow‐up studies could further consider factors such as resource availability and the need for more complex and integrated SDMs for simulation, which will be the main direction for future model development.

## 5 Conclusions

In this study, the distribution patterns of *A. glabripennis* and its host plants, *S. babylonica* and *S. matsudana* , were analyzed using an optimized MaxEnt model based on occurrence records and climatic data (current and future) of the three species. The results showed that the models exhibited an excellent fit, with AUC values exceeding 0.8. The main environmental variables affecting the potential suitable habitats of the three species were temperature and precipitation. With future climate warming and drying trends, the *A. glabripennis* and its host plants ( *S. babylonica* and *S. matsudana* ) will move to higher latitudes. In addition, future climate change will decrease the area of potential habitat for host plants. Nevertheless, *A. glabripennis* will expand more than it declines in any future climate scenario. Consequently, in the future, a combination of biological and chemical control measures should be implemented initially to regulate the population of *A. glabripennis* below the economic threshold, which is important to guide sustainable management mechanisms and early monitoring. Meanwhile, the findings of this study will contribute to a more comprehensive understanding of the biogeographic characteristics of willow and its spatial distribution pattern. This will provide theoretical guidance for the implementation of adaptive management and sustainable use of willow in the context of global change.

## Author Contributions

**Liang Zhang:** conceptualization (equal), methodology (equal), software (equal), writing – original draft (equal), writing – review and editing (equal). **Ping Wang:** funding acquisition (equal), investigation (equal), supervision (equal), writing – review and editing (equal). **Guanglin Xie:** investigation (equal), supervision (equal), writing – review and editing (equal). **Wenkai Wang:** funding acquisition (equal), supervision (equal), writing – review and editing (equal).

## Conflicts of Interest

The authors declare no conflicts of interest.

## Supporting information


Appendix S1.

## Data Availability

The authors confirm that the data supporting the findings of this study are available within the article its Supporting Information.
