# Supplementary material for: Impacts of Climate Change Conditions on the Potential Distribution of Anoplophora glabripennis and Its Host Plants, Salix babylonica and Salix matsudana, in China
Source: Ecol Evol. 2024 Dec 5;14(12):e70692. doi: 10.1002/ece3.70692 (PMC11621038; doi:10.1002/ece3.70692)
Supplement: Supplementary file 1 — Appendix S1. [file ECE3-14-e70692-s001.docx]

**Impacts of climate change** **conditions on the potential distribution of the Asian longhorned beetle, *Anoplophora glabripennis* and its host plants, *Salix babylonica* and *Salix matsudana*, in China**

Liang Zhang ^a^, Ping Wang ^a,b,*^, Guanglin Xie ^a,b^ and Wenkai Wang ^a,b,*^

^a^ *Institute of Entomology, College of Agriculture,* *Yangtze University, Jingzhou 434025, China*

^b^ *MARA Key Laboratory of Sustainable Crop Production in the Middle Reaches of the* *Yangtze River (Co-Construction by Ministry and Province), College of Agriculture, Yangtze University, Jingzhou 434025, China*

* Corresponding author at: Institute of Entomology, College of Agriculture, Yangtze University, Jingzhou 434025, China

**Supplementary Material**

**Figure S1.** Suitable habitat areas for *A. glabripennis* and its host plants (*S. babylonica* and *S. matsudana*) under different future climate scenarios.

**Figure S2.** Geographic distribution changes of *A. glabripennis* and its host plants (*S. babylonica* and *S. matsudana*) under different future climate scenarios.

**Table S1.** Suitable habitat areas of *A. glabripennis* and its host plants (*S. babylonica* and *S. matsudana*) under different climate scenarios.

**Table S2.** Alternational trends of longitude, latitude and migration distance of *A. glabripennis* and its host plants (*S. babylonica* and *S. matsudana*) in different periods.

**Table S3.** Relative change in potential range size of *A. glabripennis* and its host plants (*S. babylonica* and *S. matsudana*) under different future climate scenarios.


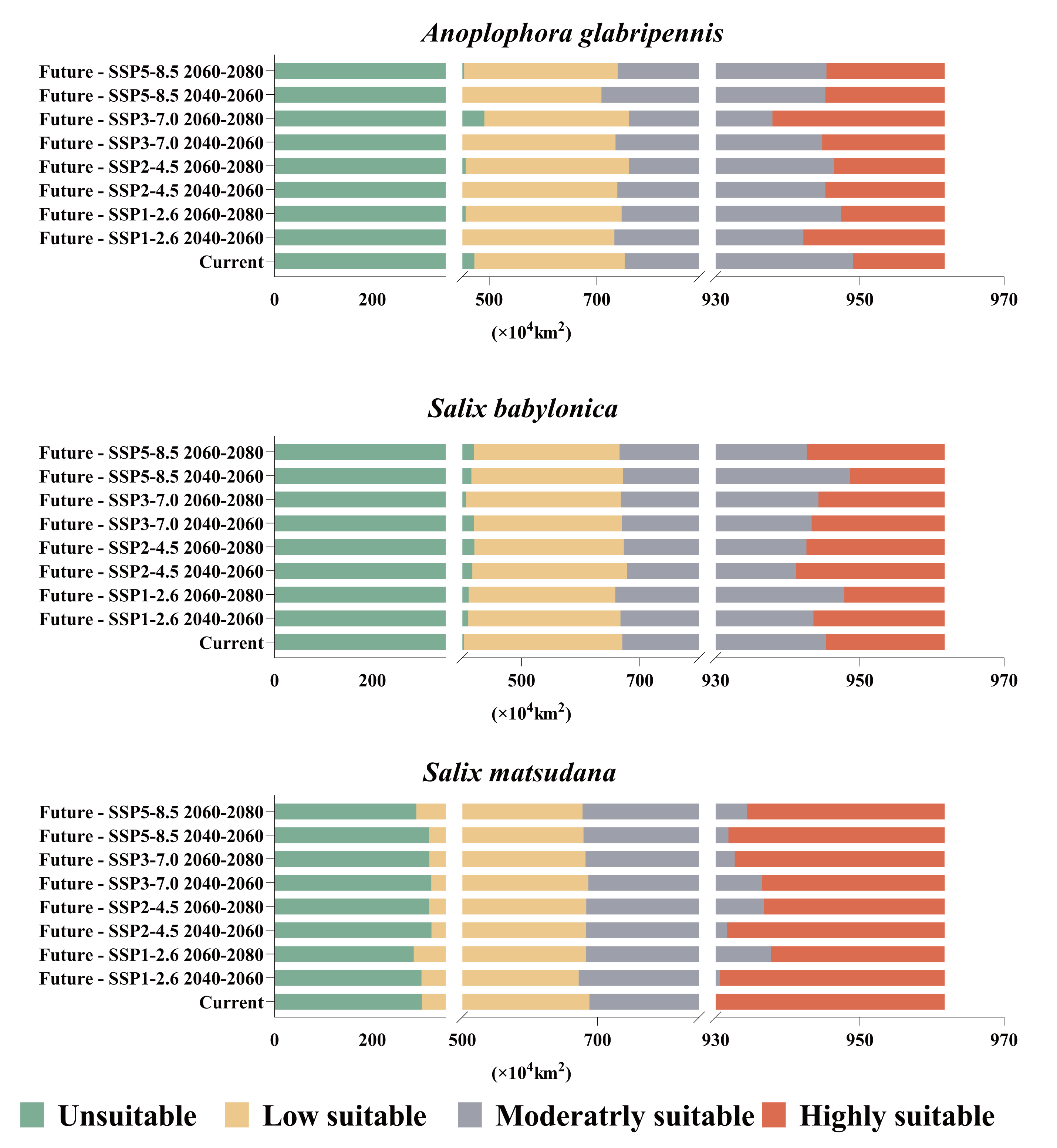


**Figure S1.** Suitable habitat areas for *A. glabripennis* and its host plants (*S. babylonica* and *S. matsudana*) under different future climate scenarios.


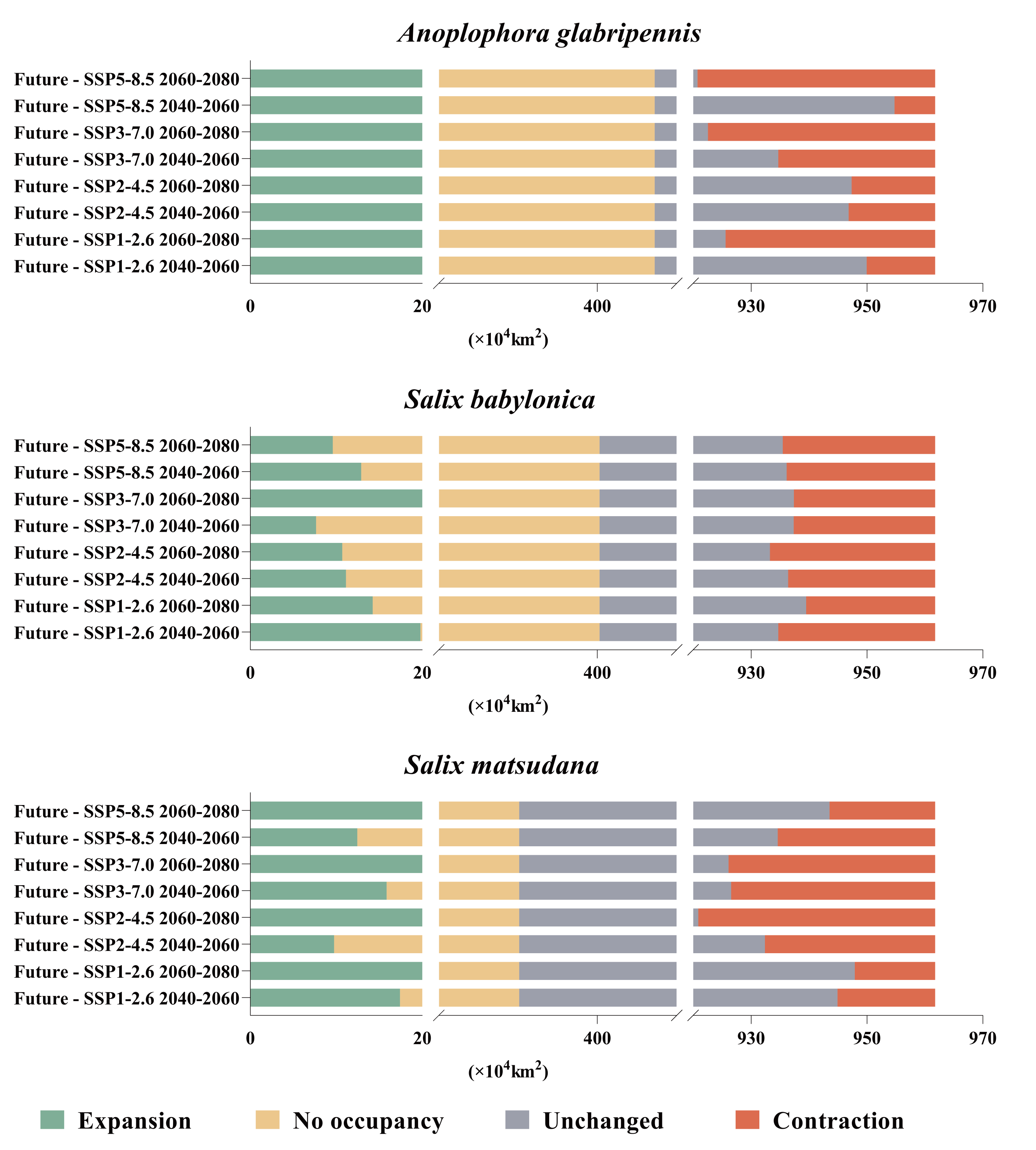


**Figure S2.** Geographic distribution changes of *A. glabripennis* and its host plants (*S. babylonica* and *S. matsudana*) under different future climate scenarios.

**Table S1.** Suitable habitat areas of *A. glabripennis* and its host plants (*S. babylonica* and *S. matsudana*) under different climate scenarios.

| Shared Socioeconomic Pathways | *S. babylonica* | | | *S. matsudana* | | | *A. glabripennis* | | |
| --- | --- | --- | --- | --- | --- | --- | --- | --- | --- |
|  | Low suitable | Medium suitable | High suitable | Low suitable | Medium suitable | High suitable | Low suitable | Medium suitable | High suitable |
| Current | 267.61 | 274.93 | 16.47 | 386.71 | 241.26 | 32.47 | 279.46 | 197.13 | 12.74 |
| Future-SSP1-2.6 2040-2060 | 257.32 | 276.20 | 18.20 | 371.49 | 258.38 | 31.13 | 308.54 | 209.43 | 19.61 |
| Future-SSP1-2.6 2060-2080 | 247.86 | 289.20 | 13.91 | 398.24 | 254.72 | 24.11 | 290.06 | 201.13 | 14.37 |
| Future-SSP2-4.5 2040-2060 | 261.29 | 262.85 | 20.61 | 361.98 | 248.66 | 30.17 | 311.52 | 206.95 | 16.55 |
| Future-SSP2-4.5 2060-2080 | 252.46 | 269.55 | 19.18 | 367.37 | 253.29 | 25.08 | 303.30 | 186.80 | 15.35 |
| Future-SSP3-7.0 2040-2060 | 250.40 | 273.40 | 18.44 | 365.63 | 250.13 | 25.34 | 292.50 | 210.00 | 16.99 |
| Future-SSP3-7.0 2060-2080 | 261.42 | 276.45 | 17.49 | 365.73 | 250.55 | 29.10 | 268.52 | 178.32 | 23.89 |
| Future-SSP5-8.5 2040-2060 | 256.01 | 277.17 | 13.11 | 363.19 | 252.51 | 30.01 | 319.68 | 236.54 | 16.55 |
| Future-SSP5-8.5 2060-2080 | 246.40 | 276.79 | 19.11 | 387.64 | 256.47 | 27.39 | 285.36 | 206.28 | 16.40 |

**Table S2.** Alternational trends of longitude, latitude and migration distance of *A. glabripennis* and its host plants (*S. babylonica* and *S. matsudana*) in different periods.

| Shared Socioeconomic Pathways | *S. babylonica* | | | *S. matsudana* | | | *A. glabripennis* | | |
| --- | --- | --- | --- | --- | --- | --- | --- | --- | --- |
|  | Longitude | Latitude | Center migration distance (km) | Longitude | Latitude | Center migration distance (km) | Longitude | Latitude | Center migration distance (km) |
| Current | 107.27 | 32.93 | - | 106.51 | 33.34 | - | 102.66 | 32.36 | - |
| Future-SSP1-2.6 2040-2060 | 106.58 | 33.90 | 125.961 | 107.24 | 33.37 | 68.01 | 104.84 | 31.36 | 234.38 |
| Future-SSP1-2.6 2060-2080 | 106.08 | 34.12 | 172.46 | 107.31 | 33.94 | 99.62 | 103.29 | 31.90 | 78.67 |
| Future-SSP2-4.5 2040-2060 | 106.07 | 34.15 | 175.32 | 105.53 | 33.60 | 96.25 | 105.56 | 31.92 | 277.76 |
| Future-SSP2-4.5 2060-2080 | 107.54 | 35.110 | 243.40 | 106.20 | 33.80 | 58.86 | 105.33 | 32.12 | 253.72 |
| Future-SSP3-7.0 2040-2060 | 107.76 | 32.82 | 47.50 | 106.92 | 33.29 | 38.70 | 103.29 | 33.15 | 106.45 |
| Future-SSP3-7.0 2060-2080 | 107.22 | 33.46 | 59.23 | 106.53 | 33.58 | 26.78 | 102.51 | 31.75 | 68.47 |
| Future-SSP5-8.5 2040-2060 | 106.96 | 35.35 | 270.13 | 105.89 | 33.64 | 66.27 | 104.37 | 32.32 | 161.09 |
| Future-SSP5-8.5 2060-2080 | 105.26 | 34.20 | 233.97 | 105.74 | 33.71 | 83.03 | 103.49 | 32.15 | 82.15 |

**Table S3.** Relative change in potential range size of *A. glabripennis* and its host plants (*S. babylonica* and *S. matsudana*) under different future climate scenarios.

| Shared Socioeconomic Pathways | *S. babylonica* | | | | *S. matsudana* | | | | *A. glabripennis* | | | |
| --- | --- | --- | --- | --- | --- | --- | --- | --- | --- | --- | --- | --- |
|  | Expansion | No occupancy | Unchanged | Contraction | Expansion | No occupancy | Unchanged | Contraction | Expansion | No occupancy | Unchanged | Contraction |
| Future-SSP1-2.6 2040-2060 | 19.80 | 382.97 | 531.93 | 27.08 | 17.43 | 283.91 | 643.58 | 16.86 | 60.07 | 412.37 | 477.51 | 11.82 |
| Future-SSP1-2.6 2060-2080 | 14.25 | 388.52 | 536.74 | 22.27 | 30.51 | 270.83 | 646.57 | 13.87 | 52.41 | 420.03 | 453.15 | 36.18 |
| Future-SSP2-4.5 2040-2060 | 11.14 | 391.63 | 533.62 | 25.39 | 9.76 | 291.58 | 631.05 | 29.39 | 60.60 | 411.84 | 474.41 | 14.92 |
| Future-SSP2-4.5 2060-2080 | 10.70 | 392.06 | 530.49 | 28.52 | 26.19 | 275.15 | 619.55 | 40.89 | 30.54 | 441.91 | 474.91 | 14.42 |
| Future-SSP3-7.0 2040-2060 | 7.66 | 395.10 | 534.58 | 24.43 | 15.87 | 285.46 | 625.22 | 35.22 | 57.23 | 415.21 | 462.26 | 27.07 |
| Future-SSP3-7.0 2060-2080 | 20.74 | 382.02 | 534.63 | 24.39 | 20.62 | 280.71 | 624.77 | 35.67 | 20.63 | 451.81 | 450.10 | 39.23 |
| Future-SSP5-8.5 2040-2060 | 12.91 | 389.85 | 533.38 | 25.63 | 12.45 | 288.89 | 633.26 | 27.18 | 90.47 | 381.98 | 482.30 | 7.03 |
| Future-SSP5-8.5 2060-2080 | 9.60 | 393.16 | 532.70 | 26.31 | 29.29 | 272.05 | 642.22 | 18.22 | 59.74 | 412.70 | 448.30 | 41.03 |
